# Homogeneous Non-Metallocene Group 4 Metals Ligated with [N,N] Bidentate Ligand(s) for Olefin Polymerization

**DOI:** 10.3390/polym16030406

**Published:** 2024-02-01

**Authors:** Zhao Wen, Changjiang Wu, Jian Chen, Shuzhang Qu, Xinwei Li, Wei Wang

**Affiliations:** SINOPEC (Beijing) Research Institute of Chemical Industry Co., Ltd., No. 14 Beisanhuan Donglu, Chao Yang District, Beijing 100013, China

**Keywords:** non-metallocene, olefin polymerization, [N,N] bidentate ligand, catalytic activity

## Abstract

The development of catalysts has significantly advanced the progress of polyolefin materials. In particular, group 4 (Ti, Zr, Hf) non-metallocene catalysts ligated with [N,N] bidentate ligand(s) have garnered increasing attention in the field of olefin polymerization due to their structurally stability and exceptional polymerization behaviors. Ligands containing nitrogen donors are diverse and at the core of many highly active catalysts. They mainly include amidine, guanidinato, diamine, and various N-heterocyclic ligands, which can be used to obtain a series of new polyolefin materials, such as ultrahigh molecular weight polyethylene (UHWMPE), olefin copolymers (ethylene/norbornene and ethylene/α-olefin) with high incorporations, and high isotactic or syndiotactic polypropylene after coordination with group 4 metals and activation by cocatalysts. Herein, we focus on the advancements and applications of this field over the past two decades, and introduce the catalyst precursors with [N,N] ligand(s), involving the effects of ligand structure, cocatalyst selection, and polymerization conditions on the catalytic activity and polymer properties.

## 1. Introduction

Polymer materials are increasingly widely used in people’s daily lives. Polyolefins have become an indispensable part of the polymer field, and now they are also the most used and diverse polymer materials [1,2]. Polyolefins have the characteristics of excellent mechanical strength, chemical inertness, recyclability, and electrical insulation. This makes them very cost-effective. Polyolefins are mainly used in economic pillar industries, such as packages, construction, automobiles, sports, electronics, and agriculture. In 2021, global plastic production exceeded 390 million tons, among which polyolefin dominated by polyethylene and polypropylene accounted for 46.2%. The aforementioned statements suffice to substantiate its significance. In the field of polyolefins, particularly in relation to high-end materials such as UHMWPE, polyolefin elastomer, and cycloolefin copolymer, these materials exhibit enhanced economic value and utility. However, the synthesis of such advanced materials and the exploration of novel alternatives heavily rely on advancements in high-performance catalyst research.

It has been over sixty years since Ziegler and Natta discovered the polymerization catalysts and first obtained high-density polyethylene (HDPE) and isotactic polypropylene (iPP). During this time, different types of catalysts such as Phillips chromium-based catalysts, metallocene catalysts, and non-metallocene catalysts made significant breakthroughs in the field of polymer catalytic synthesis [3,4,5,6]. In the 1980s, the milestone progress of metallocene catalysts had a significant impact on polymer synthesis and catalytic chemistry, due to the discovery of methylaluminoxane (MAO), a high-efficiency cocatalyst, which led to a revolution in polyolefin industry. The most significant difference between metallocene catalysts and Z-N catalysts is the single-site characteristic of the former. However, there are some problems existing with the use of metallocene catalysts in olefin polymerization: (i) the high cost of synthesis; (ii) the high cost of the large dosage of MAO to scavenge impurities to maintain high activity; (iii) at the same time, the patent protection of the catalyst structure is too comprehensive, making it difficult to achieve innovation in metallocene structures. Therefore, researchers turned their attention to non-metallocene catalysts that can also form a single active site in the catalytic process by replacing cyclopentadienyl with other organic groups [7].

**Figure 1:**
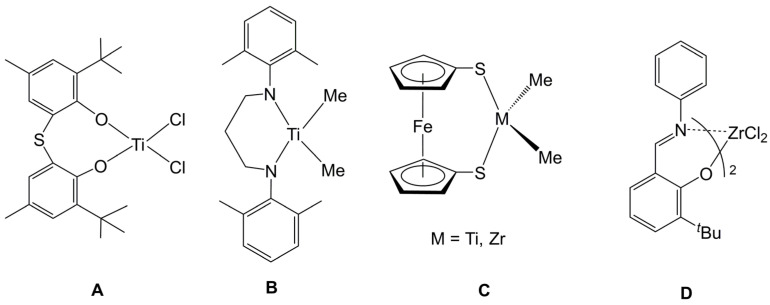
The structure of complexes (**A**–**D**).

Non-metallocene catalyst precursors, in a narrow sense, are defined as the organometallic compounds of group 4 metals (Ti, Zr, and Hf) without a cyclopentadienyl group. These catalysts are one of the hot topics in the research into olefin coordination polymerization because of their diverse ligand designs and strong copolymerization ability. The development of non-metallocene catalysts was initiated in the mid-1990s. In 1995, Schaverien et al. [8] reported that biphenyloxide titanium complex **A** (complexes **A**, **B**, **C** and **D** see Figure 1) showed a relatively high activity for olefin polymerization. In 1996, McConville et al. [9] reported a chelated diamide titanium complex **B**, which can polymerize α-olefin at room temperature. In 1999, Gibson et al. [10] reported titanium and zirconium complexes **C** with a ferrocenyldithiolate ligand leading to medium to low activity toward ethylene polymerization. Although new non-metallocene catalysts had been developed, their activity was always lower than the typical metallocene catalyst Cp_2_ZrCl_2_ [11]. In 2000, Fujita group, Mitsui Chemical Company, reported a non-metallocene olefin catalyst with phenoxy-imine as bidentate ligand and zirconium as the metal center, the so-called “FI catalyst” **D** exhibiting very high activity (51.9 × 10^6^ g-PE/mol-Zr·h) twenty times that of Cp_2_ZrCl_2_. By adjusting the polymerization conditions and changing the ligand structure, the activity can be further increased to more than two orders of magnitude greater than Cp_2_ZrCl_2_ [7,12]. By using ^13^C NMR spectroscopy, they revealed that the syndiotacticity of polypropylene arose from a chain-end control mechanism, indicating that the propylene monomer included chain propagation by 2,1-insertion [13]. The syndiospecificity can be attributed to a rapid site-inversion relative to chain propagation if a catalytically active species possesses an octahedral geometry with a *trans*-O and *cis*-N arrangement. In addition, when changing the structure of ligands (monoamidinate and pyridine-amido ligands) or replacing the substituents (hydrogen on the imine carbon is replaced by the large steric hindrance substituent [14]), the mechanism of polymerization is changed, and finally iPP is obtained. So, practically all possible combinations of regiochemistries and stereochemistries have been observed in propylene polymerization with non-metallocene catalysts [15].

Non-metallocene catalysts have been extensively researched and elaborated upon by numerous scholars up to this point. For example, in 2003, Gibson et al. [16] reviewed the application and development of non-metallocene catalysts in olefin polymerization according to the classification of the coordination metal center, and the importance of high-throughput screening (HTS) for the study of catalysts was proposed. In 2011, Kim et al. [17] reviewed the synthesis of non-metallocene catalysts based on N-heterocyclic ligands and their activity in olefin oligomerization and polymerization over the previous 25 years. In 2020, Sun et al. [18] reviewed the advancements of non-metallocene catalysts coordinated with N-ligated for (co)polymerization of ethylene. However, many reviews lack detailed reports on the types and applications of bidentate non-metallocene catalysts coordinated with group 4 metals using N-donors. Therefore, our focus is on summarizing ligands with [N,N] and the structures of non-metallocene catalysts featuring group 4 metals (Ti, Zr, and Hf) as central metal atoms. We emphasize the activity and applications of catalytic systems with different structures in olefin (co)polymerization while also discussing the future development trends of [N,N]-bidentate non-metallocene catalysts.

## 2. [N,N]-Ligands Metal Complexes

[N,N]-ligands metal complexes are obtained by coordinating or directly forming covalent bond connections between two *N*-donors on the ligand group and group 4 metals (Ti, Zr, and Hf). These complexes are the most abundant and widely used bidentate non-metallocene catalysts. According to their different ligand structures, [N,N]-ligands are composed mainly of amidine, guanidinato, diamine, and various N-heterocyclic ligands.

### 2.1. Amidinate Ligands Metal Complexes

**Figure 2:**
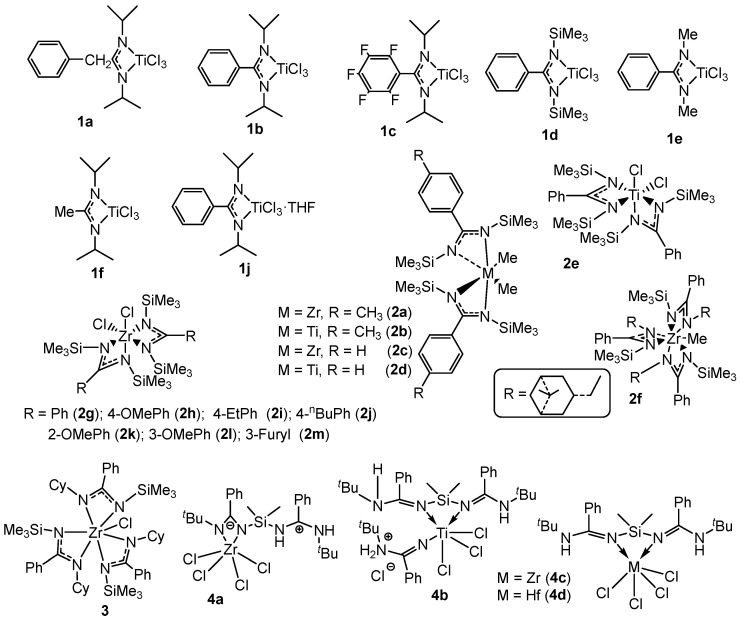
The structure of complexes **1a**–**1j**, **2a**–**2m**, **3**, **4a**–**4d**.

**Scheme 1:**
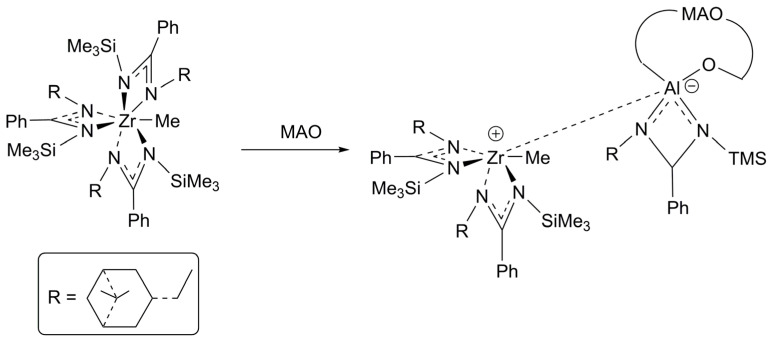
The reaction of the tri-amidinate complex’s migration to aluminum in MAO.

In the amidinate ligands, the anionic moiety [R-C(NSiMe_3_)_2_]^−^ is a four-electron donor, promoting higher electrophilicity at the metal center. Within the amidinate-based ligands, a variety of benzamidinate group 4-containing complexes have been described. Benzamidine ligand is considered to be spatially equivalent to cyclopentadienyl (Cp) and its derivatives, which lead the polymerization of various olefin monomers to be carried out. Zambelli et al. [19,20] reported a series of monoamidinate titanium complexes **1a**–**1g** (complexes **1**–**4** see Figure 2), which can be activated by MAO and used as precatalysts in propylene polymerization at low temperatures (−60 °C). In comparison to **1a**, **1b**, **1d**, and **1g**, the **1c**/MAO catalytic system has a larger yield and higher *M*_n_. The stereochemical triad compositions and the amounts of vicinal methyls of **1e** and **1f** showed that these products were prevailingly isotactic and more regioregular than others. Detailed polymerization data are shown in Table 1. These complexes/MAO catalysts systems can achieve stereospecific polymerization of 1,3-butadiene and styrene. The monoamidinate ligand complexes, however, are unstable, leading to the deactivation of metal centers during high-temperature olefin polymerization. Eisen et al. [21,22,23,24] reported the *C*_2_-symmetric bis(benzamidinate) dichloride or dialkyl complexes **2a**–**2m** of group 4 metals. Among them, complexes **2a**–**2d**, activated with MAO, can yield different species and properties of polypropylene, for example, the **2a**/MAO catalytic system can obtain atactic polypropylene at atmospheric pressure in toluene, **2d**/MAO can obtain elastomer with polypropylene, and the **2d**/MAO catalytic system had the highest activity (28.5 × 10^5^ g-PP/mol-Ti·h). A cationic bis-amidinate dialkyl complex **2f**, resulting from ligand dissociation of the tri-amidinate complex and its migration to aluminum in MAO, produced elastomeric polypropylene, as illustrated in Scheme 1. The **2a**, **2b**, and **2f**/MAO catalytic systems can effectively polymerize 1,5-hexadiene, with the highest catalytic activity from **2f**/MAO (11.0 × 10^4^ g-polymer/mol-Zr·h). Complexes **2g**–**2m** with different aryl substituents had moderate activity, and due to the ligand dissociation, the molecular weight distribution (MWD) of polypropylene became wider. For a more intuitive comparison, the polymerization data of mono-amidinate and bis(amidinate) complexes are shown in Table 2. Zhou et al. [25] reported that asymmetric aliphatic benzamidine zirconium complex **3** can be used for ethylene polymerization, exhibiting a high activity of 54.4 × 10^4^ g-PE/mol-Zr·h. This method of combining asymmetric aromatic and chiral amidine ligands provides ideas for the design of new ligands. In addition, novel structural catalysts can also be obtained by bridging with heteroatoms. Liu et al. [26] reported that alkyl-ended silicon-bridged *ansa*-bis(amidinate) complexes **4a**–**4d** could also be used for ethylene polymerization after MAO activation, but their activity was relatively lower than **3**/MAO. This may be because the bridge structure affected the original steric hindrance of the catalyst. It was found that in mono-amidinate ligand complexes, syndiotactic polypropylene and polystyrene were obtained, and catalytic activity was increased due to the perfluorophenyl substituent of the amidinate ligand increasing the electrophilicity of the active species. In comparison, bis-amidinate ligand complexes could obtain poly(methyl-1,3-cyclopentane) and isotactic, atactic, and elastomeric polypropylene, and complexes with oxygen-containing ligands showed higher activities than complexes with phenyl or alkyl benzene rings, though the activity was moderate. Higher activity and better stereoregularity were achieved by performing the polymerization in CH_2_Cl_2_, which had higher polarity compared with toluene. Additionally, the bis(benzamidinate) zirconium complex with a methyl group in the *para*-position of the benzene ring can obtain highly isotactic polypropylene with high activity, but, when titanium was used as the metal center without any substituents on the benzene ring, both activity and isotacticity decreased, resulting in the formation of elastomeric polypropylene. In the zirconium complexes, the more open metal center could possibly allow for more facile propylene insertion.

### 2.2. Guanidinato Ligand Metal Complexes

**Figure 3:**
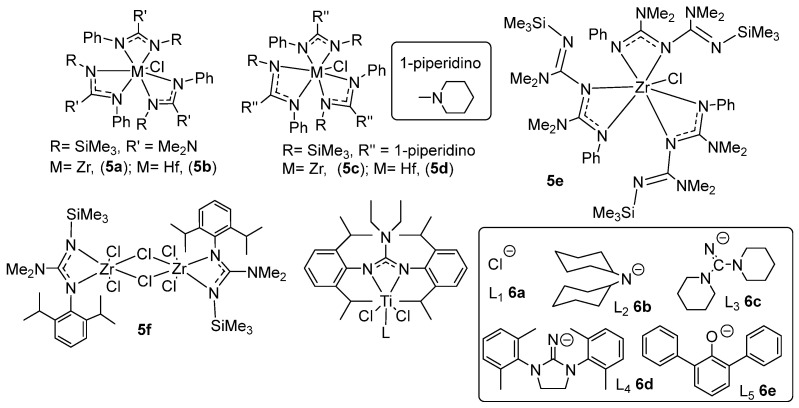
The structure of complexes **5a**–**5f**, **6a**–**6e** (L**_1_**–L_5_).

Compared with amidino ligands, guanidine ligands also have strong π-electron donating abilities and can ligate with central metal as neutral, mono, and multiple anionic forms. So, the complexes with guanidinato ligands have potential for coordination and organometallic chemistry and could be used as alternatives to unique cyclopentadienyl species in order to form suitable catalytic sites for the polymerization of α-olefins. Liu et al. [28,29,30] reported non-symmetric guanidinato zirconium and hafnium complexes **5a**–**5f** (complexes **5** and **6** see Figure 3). Among them, complexes **5a**–**5e** showed moderate activity and high molecular weight (**5d**/MAO activity was 29.2 × 10^3^ g-PE/mol-Hf·h, *M_η_* = 3.5 × 10^5^) in ethylene polymerization. This can be attributed to the strong donor substituents (more basic) and guanidinato ligand increasing the electron density of the metal. Furthermore, it was found that the molecular weight of PE increased with the increase in polymerization temperature. When the alkyl substituent in the *ortho* position of phenyl connected to the amino, the triguanidinato complex was no longer produced, and the asymmetric chlorine-bridged guanidinato binuclear zirconium complex **5f**, which was a high-activity ethylene polymerization catalyst system (**5f**/MAO, activity was 64.8 × 10^4^ g-PE/mol-Zr·h), was obtained by selecting the appropriate Al/Zr (molar ratio of co-catalyst to complex). The increase in the number of cation centers after the activation of the binuclear complex facilitated polymerization, thereby enhancing its catalytic activity. Under similar conditions, comparing the activity of various catalysts, it was found that the activity of the non-symmetric guanidinato complexes was not significantly affected by the change of substituents or central metals. However, when a second monoanionic ligand was introduced to the monoanionic guanidinato ligand, the behaviors of the complex changed significantly. Kempe et al. [31] reported relevant complexes **6a**–**6e**. The guanidinato titanium dichloride complexes **6b**–**6e** were prepared by simple salt-elimination reactions from the titanium precursor **6a** and the Li salt of the ligands. Among them, complex **6b** was completely inactive, whereas other complexes with bulky monoanionic bidentate N-ligands had high activity of ethylene polymerization. The addition of different bulky ligands led to different polymerization products, for example, complex **6d**, which was activated by dry methylaluminoxane (d-MAO) to obtain UHMWPE (*M*_w_ = 24.88 × 10^5^ g/mol) with high activity (88.0 × 10^5^ g-PE/mol-Ti·h). In view of the properties of the amidino and guanidine ligands of the monoanion we could speculate that the many bulky monoanionic non-cyclopentadienyl ligands might be useful candidates to further develop nonbridged, mixed-ligand group 4 metal complexes combining high polymerization activity and unusual selectivity patterns.

### 2.3. Diamine Ligand Metal Complexes

Diamine-based non-metallocene catalysts have a small number of electrons, high electrophilicity, and higher catalytic activity. In this review, ligands with β-diiminato, α-diiminato, and amino-containing complexes (amino groups directly attached to imido, or between N, P, Si, and ferrocene), are collectively classified as diamine ligand complexes.

**Figure 4:**
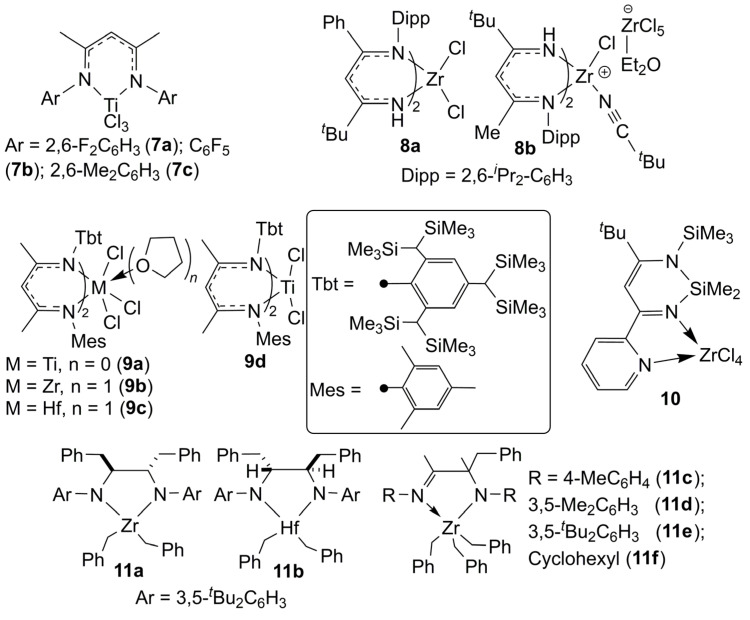
The structure of complexes **7a**–**7c**, **8a**–**8b**, **9a**–**9d**, **10**, **11a**–**11f**.

**Scheme 2:**
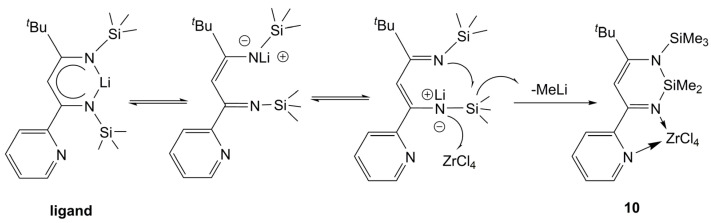
Plausible mechanism for the formation of complex **10**.

β-diiminato ligands appeared early in the field of olefin polymerization, have a wide range of applications, and have diverse ligand structures. Xie et al. [32] reported a series of novel mono β-diiminato titanium complexes **7a**–**7c** (complexes **7**–**11** see Figure 4), and the significant electronic effects of fluoro-substituents on the olefin polymerization activity of mono β-diiminato titanium complexes were identified. Titanium complex **7a** with fluorine-containing β-diiminato ligands, on activation with modified methylaluminoxane (MMAO), had very high activity for polymerization of ethylene (76.8 × 10^5^ g-PE/mol-Ti·h) and higher activity for copolymerization of ethylene/1-henxene (26.1 × 10^6^ g-copolymer/mol-Ti·h). During copolymerization, the activity increased with the increase in the initial concentration of 1-hexene, which showed the positive “comonomer effect”. The high activity of such o-fluoro-substituted complexes was due to the non-covalent interaction between o-fluoro atoms and ethylene monomers that contributed to the coordination of ethylene with the central metal [33], thereby improving catalytic activity. In addition to mono β-diiminato ligands, Sun et al. [34] reported asymmetric bis(β-diiminato) zirconium **8a** and **8b**, which can also be used for ethylene polymerization, and under the condition of MAO activation and appropriate increase of ethylene pressure (10 atm), the activity of **8a**/MAO was 10.0 × 10^5^ g-PE/mol-Zr·h. Comparing mono and bis β-diiminato ligands, the activity of **8a**/MAO is not as good as that of the **7a**/MMAO catalytic system, but a high molecular weight of PE can also be obtained (*M*_w_ = 26.0 × 10^5^ g/mol). This is due to the increase of steric hindrance around the active metal center leading to β elimination and the decrease of chain transfer reaction rate. According to this theory, when there are substituents with larger steric hindrance on the N donor, ultrahigh molecular weight polymers will be obtained. Tokitoh et al. [35] reported β-diiminato complexes **9a**–**9d** with extremely bulky substituents, in which Tbt-substituted (Tbt = 2,4,6-[(Me_3_Si)_2_CH]_3_C_6_H_2_) complex **9a**, activated by Al(*i*-Bu)_3_/[PhNHMe_2_][B(C_6_F_5_)_4_], had high activity in ethylene polymerization (65.0 × 10^5^ g-PE/mol-Ti·h) and ethylene/1-hexene polymerization (51.0 × 10^5^ g-polymer/mol-Ti·h). Inspired by co-activation of cocatalysts, corresponding precatalysts can also have the same innovation. Zhang et al. [36] successfully prepared medium-molecular-weight branched PE by using a novel binary tandem catalytic system composed of 2,6-bis(imino)pyridyl iron complex and mono β-diiminato titanium complex **7a**. After adjusting the molar ratio of Fe and Ti (Fe:Ti = 4:1), the activity of **7a**/MMAO was up to 52.3 × 10^5^ g-PE/mol-Ti·h. Chen et al. [37] reported complex **10**, having high activity in ethylene polymerization (22.4 × 10^4^ g-PE/mol-Zr·h) after activation by MAO, which was obtained by the neutral cyclo-1,3-diazasilane heterocyclic ring coordinated with the zirconium center, different from the traditional coordination mode of β-diiminato (a plausible mechanism for the formation of **10** is shown in Scheme 2). The formation of **10** in the reaction of the ligand with ZrCl_4_ was presumably an intermolecular interaction of the weak acceptor, zirconium, with the anionic imido nitrogen leading to the cleavage of a Si–C bond and the formation of a new Si–N bond with concomitant elimination of MeLi. The (co)polymerization results of β-diiminato complexes are shown in Table 3. It was found that the metal center had a wide open coordination environment in mono-/bis(β-diminiato) complexes, which caused a little steric hindrance for the coordination of the comonomer and metal center, thus obtaining high catalytic activity.

The structure of α-diimine ligands is similar to that of the β-diiminato ligand, and part of α-diimide catalyst is obtained by alkylation of a neutral ligand with a C=N bond; this method can replace the elimination of salts, amines, and alkanes to synthesize catalysts. Mashima et al. [38] reported α-diimine complexes **11a**–**11f** with moderate polymerization activity for 1-hexene and vinylcyclohexane and copolymerization of ethylene and 1-hexene, in which the **11b**/[Ph_3_C][B(C_6_F_5_)_4_] catalytic system could obtain narrow molecular-weight distribution and high-molecular-weight polymer (*M*_w_ = 4.51 × 10^5^, MWD = 1.5). Comparing α-diimide and β-diiminato complexes, the former have been the subject of fewer reports and studies. This is due to the weak structural stability of the five-membered ring formed by α-diimide complexes with metal centers; the raw materials for the synthesis of ligands are relatively scarce and α-diimide titanium complexes could not be formed because of the small ionic radius of titanium.

**Figure 5:**
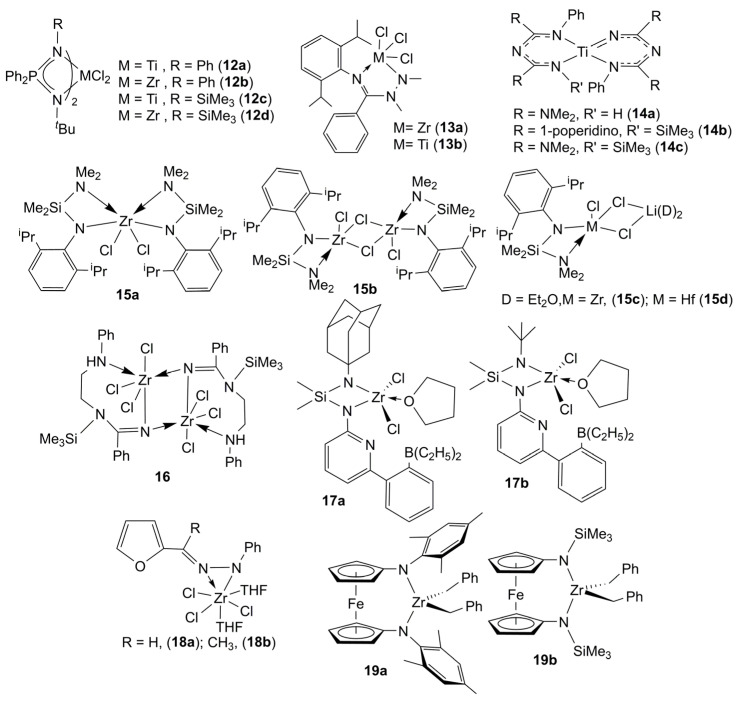
The structure of complexes **12a**–**12d**, **13a**, **13b**, **14a**–**14c**, **15a**–**15d**, **16**, **17a**, **17b**, **18a**, **18b**, **19a**, **19b**.

Amino-containing complexes exhibit exceptional performance and possess a unique structural arrangement. In comparison to β-diiminato complexes, these compounds require a lengthier synthetic pathway. However, their innovative structures broaden the scope of non-metallocene catalysts, with some demonstrating high activity in olefin polymerization. Zhang et al. [39] reported titanium and zirconium complexes **12a**–**12d** (complexes **12**–**19** see Figure 5) with aminoiminophosphorane ligands and speculated that at higher monomer concentrations (ethylene pressure of 0.5 MPa), the coordination between the active species and monomer will be more effective and the steric congestion could be negligible. Bildstein et al. [40] reported complexes **13a** and **13b** with [N,N]-iminohydruoxamato ligands. The **13a**/MAO catalytic system had moderate activity in ethylene polymerization (30.7 × 10^4^ g-PE/mol-Ti·h). The structural stability of these catalysts was weak, which hindered olefin coordination. Zhou et al. [41] reported titanium imido complexes **14a**–**14c** with dianionic ligands of triazapentadienyl derivatives. The **14a**/MAO catalytic system had a high catalytic activity (56.6 × 10^3^ g-PE/mol-Ti·h), and the complex structure was more stable than **13a** and **13b**. Sun et al. [42] reported a series of complexes **15a**–**15d** of *μ*_2_,*η*^1^-*N*-[(*N*,*N*-dimethylamino)dimethylsilyl]-2,6-diisopropylanilido. The silane-bridged pseudotetrahedral complex **15a** and chloro-bridged binuclear tetrahedral complexes **15b**–**15d**, activated by MAO, exhibited high activity for ethylene polymerization (**15c**/MAO catalytic system with the highest activity up to 17.3 × 10^5^ g-PE/mol-Zr·h), and obtained PEs with broad MWD (4.2–65). Complex **15a** obtained polymer with the narrow MWD and relatively low *M*_w_, attributing to the two ligands occupying spaces around zirconium influencing the slow coordination of ethylene, and binuclear complexes **15b**–**15d** seem to form less stable active species. Although activity was higher, multiple active sites were formed, leading to PEs with wider MWD. In addition, complexes **15a** and **15b** could be used in copolymerization of ethylene and 1-hexene, and **15b**/MAO had high activity (10.3 × 10^5^ g-copolymer/mol-Zr·h). Liu et al. [43] reported an amidino–amino binuclear zirconium complex **16**, the formation of which was due to the presence of trimethylsilane that extended the skeleton of the ligand and formed a more stable seven-ring coordination. Meanwhile, **16**/MAO exhibited high catalytic activity (48.6 × 10^4^ g-PE/mol-Zr·h), a high molecular weight, and broad distribution (*M*_w_ = 9.5 × 10^5^g/mol, MWD = 10.08). This is closely associated with the enhancement of zirconium electrophilicity and the presence of binuclear species. Upon examining the aforementioned structures of these complexes, it can be observed that coordination complexes with double ligands exhibit improved stability and lead to polymers with a narrow MWD, while coordination complexes with single ligands demonstrate high activity and form polymers with a relatively wide MWD. Additionally, binuclear complexes result in polymers with an even wider MWD. These enable the design of the catalyst structure to be further optimized based on the desired properties of the polymer product, thereby enhancing efficiency in designing a rational catalyst structure.

Buchmeiser et al. [44] reported novel zirconium complexes **17a** and **17b** with a 6-[2-(diethylboryl)phenyl]pyrid-2-ylamido motif. Complexes **17a** and **17b** had high catalytic activity and could obtain ethylene/cyclopentene (E/CPE) copolymer (75.0 × 10^5^ g-copolymer/mol-Zr·h) and high-molecular-weight linear HDPE (activity = 18.8 × 10^4^ g-PE/mol-Zr·h, 1.8 × 10^6^ g/mol ≤ *M*_n_ ≤ 4.0 × 10^6^ g/mol). The coordination between CPE and metals facilitated alkyl transfer to the aluminum atoms of MAO, resulting in the copolymer lacking vinyl end groups. In addition, a comparison between complex **17a** and **17b** revealed that the presence of a bulky substituent in close proximity to the metal center resulted in ultra-high molecular weight at 90 °C. This was due to the large substituent group effectively impeding both α- and β-agostic interactions and preventing any of the β-hydride elimination reactions, which made chain propagation continue. Sun et al. [45] reported hydrazonide complexes **18a** and **18b**. These complexes were highly sensitive to air and moisture due solely to using less bulky hydrazonide ligands. After MMAO activation, complex **18a** showed moderate activity (36.0 × 10^3^ g-PE/mol-Zr·h) and produced UHMWPE with narrow distribution (*M*_w_ = 2.05 × 10^6^ g/mol, MWD = 1.49). Arnold et al. [46,47] described ferrocene-containing olefin polymerization zirconium complexes **19a** and **19b**; after [Ph_3_C][B(C_6_F_5_)_4_](TB) activation, **19a**/TB could be used in the polymerization of 1-hexene and yielded poly-1-hexene with molecular weights of 20,000 and narrow MWD (1.3–1.4). This was due to the CH activation of a mesityl methyl group in this catalytic system, which may lead to deactivation. After changing the substituents on the amido, the **19b**/TB catalytic system had low activity for ethylene polymerization (10.2 × 10^4^ g-PE/mol-Zr·h). While ferrocene-containing complexes had less activity for olefin polymerization, the increased stability facilitates the investigation of their structure and reactivity. The polymerization results of amino-containing complexes are shown in Table 4. It was found that the novel amino-containing complexes, particularly **17a** and **18a**, exhibited relatively moderate activity; however, their distinctive ligand structure enabled the synthesis of UHMWPE with MWD below 10 through homopolymerization of ethylene.

### 2.4. N-Heterocyclic Amido Ligand Metal Complexes

N-heterocyclic ligands play a crucial role in [N,N]-based non-metallocene catalysts, serving as essential components. The metal complexes exhibit diverse structures and steric properties owing to the presence of different nitrogen heterocyclic coordination, thereby influencing their catalytic activity in olefin polymerization. According to the different nitrogen heterocycles, they are mainly divided into pyrrolide-, pyridine-, pyrazolato-, thiazole-, imidazole-, and quinoline-amido ligands.

**Figure 6:**
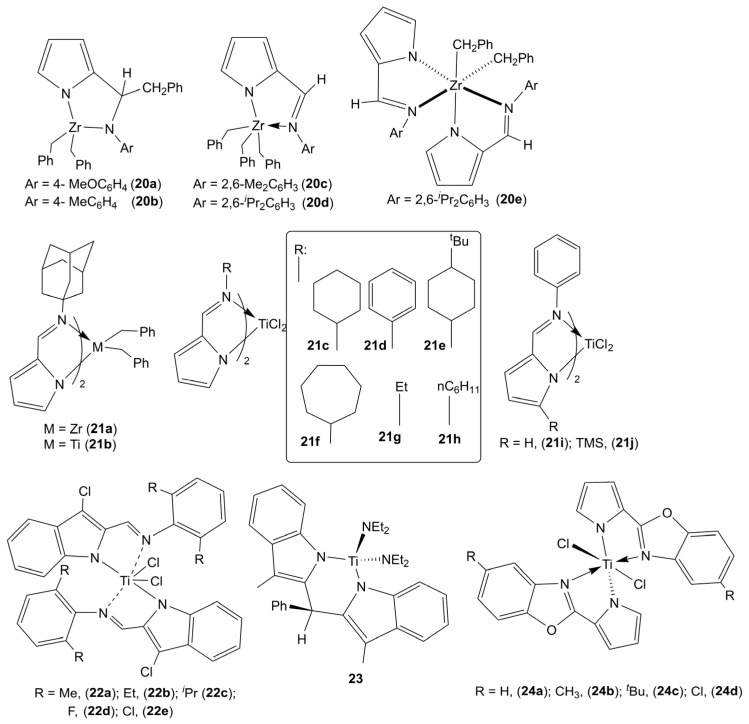
The structure of complexes **20a**–**20e**, **21a**–**21j**, **22a**–**22e**, **23**, **24a**–**24d**.

**Figure 7:**
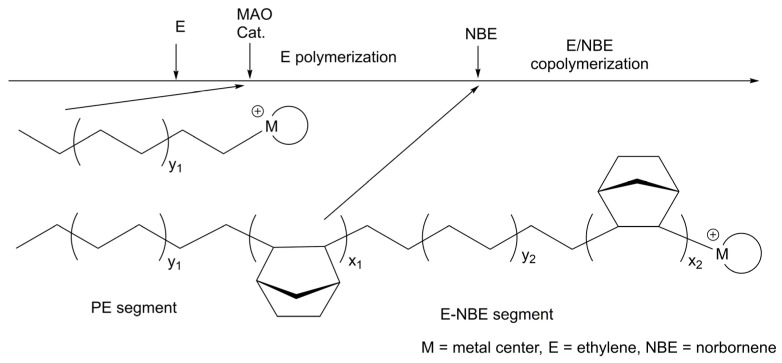
The synthesis of the block copolymer.

**Figure 8:**
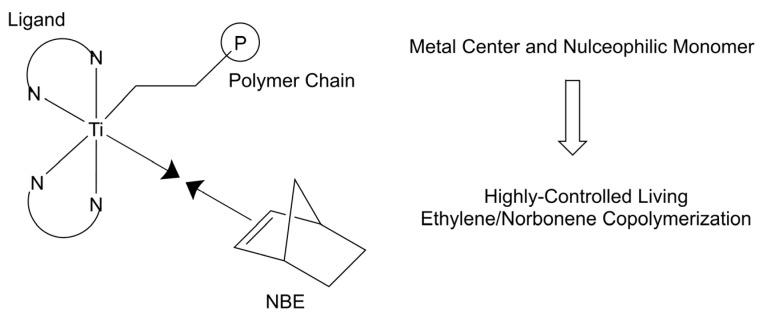
The electronic attractive interaction of PI catalysts.

Mashima et al. [48] reported benzyl zirconium complexes **20a**–**20e** (complexes **20**–**24** see Figure 6) of 2-(*N*-aryliminomethyl)pyrrolyl ligand. The **20b**/MMAO catalytic system had very high activity of ethylene polymerization at 0 °C (10.8 × 10^5^ g-PE/mol-Zr·h), whereas **20c**–**20e**, which had the di-/tribenzyl zirconium center supported by one (iminomethyl)pyrrolyl ligand, exhibited 10^2^ less catalytic activity. The results showed that the activity of the catalyst system could be improved by alkylation of the imino moiety of the ligand in the pyrrolide-imine ligand. The group 4 metal centers coordinated with bis(pyrrolide-imine) ligand, which were developed by Fujita’s team in the early 2000s, were often referred to as PI catalysts. In comparison to FI catalysts, the catalytically active cationic species originating from PI catalysts exhibited higher electrophilicity and a sterically open nature, enabling strong electron interaction with olefin monomers and influencing the activity of the catalyst. As a consequence, Fujita et al. reported [49,50,51] different structures of pyrrolide-imine complexes **21a**–**21f**. PI catalysts had high activity for ethylene polymerization, among which the **21a**/MAO catalytic system, with an adamantyl substituent, had the highest activity (22.9 × 10^6^ g-PE/mol-Zr·h) after exchanging the substituent on the imine with cyclohexyl. The **21c**/MAO catalytic system had high activity (42.7 × 10^5^ g-polymer/mol-Ti·h) for the copolymerization of ethylene and norbornene (NBE); the copolymers had narrow MWD (1.28) and different kinds of block copolymers were obtained. The block copolymers were synthesized using a sequential-addition polymerization procedure (Figure 7). Additionally, in the **21c**/MAO catalytic system, the *M*_n_ value increased proportionally with polymerization time (1 min, *M*_n_ = 74 × 10^3^; 3 min, *M*_n_ = 179 × 10^3^; 5 min, *M*_n_ = 285 × 10^3^; 10 min, *M*_n_ = 521 × 10^3^), and the narrow *M*_w_/*M*_n_ value was retained (*M*_w_/*M*_n_ 1.07–1.16), suggesting that the system was indeed living. Calculation via DFT confirmed that when ethylene was connected to the metal center in the copolymerization, the next inserted molecule was more inclined to NB because the coordination energy was −17.05 kg/mol for NBE and −8.72 kg/mol for ethylene, so this living copolymerization was highly controllable. This highly controllable living copolymerization based on the active species of PI catalysts possessed high affinity and high incorporation ability for NBE and PI catalysts therefore had an electronic attractive interaction (Figure 8). In addition, **21c**/MAO had high catalytic activity for ethylene/propylene copolymerization (13.1 × 10^5^ g-polymer/mol-Ti·h) and ethylene/1-hexene copolymerization (45.8 × 10^5^ g-polymer/mol-Ti·h).

Based on these advantages of pyrrolide-imino ligands, a series of derivatives of pyrrolide-imino ligands can be obtained by modifying the structure of the ligand, which could also be utilized in olefin polymerization when coordinated with group 4 metals with excellent properties. Sun et al. [52] reported that bis(imino-indolide) titanium complexes **22a**–**22e**, activated by MAO, could be used in ethylene polymerization and E/NBE copolymerization to obtain polymers of high molecular weight, and found that titanium complexes with electron-withdrawing groups in ligands had higher catalytic activity than complexes with electron-donating groups (detail polymerization data are shown in Table 5). Ohta et al. [53] reported di(methylindolyl)-phenylmethane titanium complex **23**. The ethylene polymerization activity of the **23**/MMAO catalyst system was low (16.3 × 10^3^ g-PE/mol-Ti·h), but pretreatment of **23** with ClSiMe_3_ followed by activation with MMAO improved the activity up to 15.4 × 10^4^ g-PE/mol-Ti·h. ClSiMe_3_ served as a chlorinating reagent, and the substitution of the chloride ligand with an alkyl ligand via MMAO occurred at a faster rate than that of the amido ligand, resulting in improved ethylene polymerization activity. In addition to indolide, pyrrolide-imine derivatives also have pyrrolide-benzoxazole ligands. You et al. [54] reported the titanium catalysts **24a**–**24d** supported by pyrrolide-benzoxazole ligands and their application in ethylene polymerization. After activation with MAO, all complexes were able to obtain high-molecular-weight PE (96.10 × 10^5^ g/mol) with high activity, in which the maximum activity was 39.8 × 10^4^ g-PE/mol-Ti·h when there was chlorine substitution in the benzene ring of the ligand framework. Because the electron-withdrawing ability of chlorine increases the electrophilicity of the metal center, it thus promotes the coordination of ethylene.

**Figure 9:**
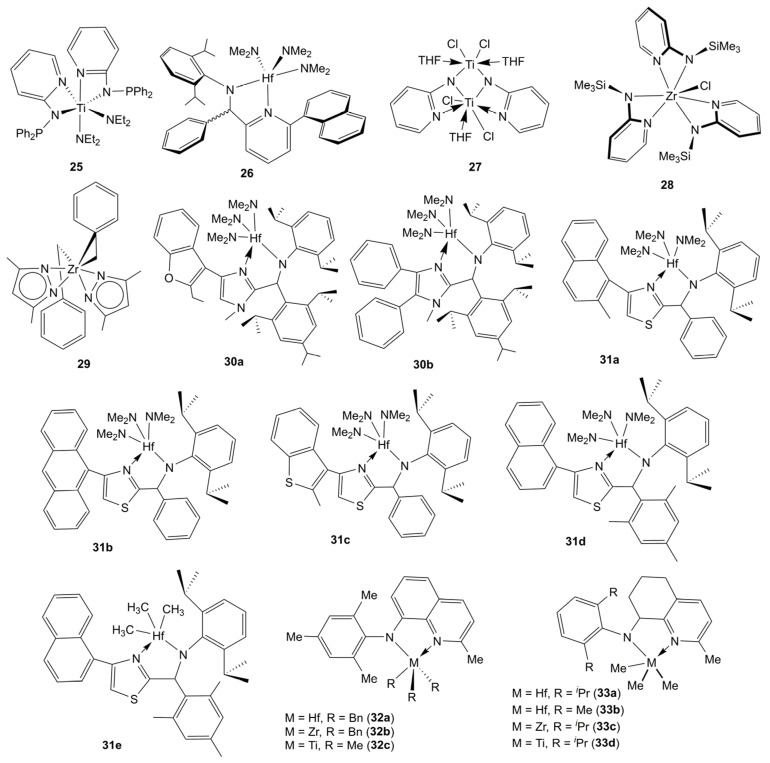
The structure of complexes **25**, **26**, **27**, **28**, **29**, **30a**, **30b**, **31a**–**31e**, **32a**–**32c**, **33a**–**33d**.

Pyridine-amido ligands have relatively simple synthesis pathways and easily regulated structures. Eisen et al. [55] reported complex **25** (complexes **25**–**33** see Figure 9), which had good stability and moderate catalytic activity for propylene polymerization (13.1 × 10^3^ g-PP/mol-Ti·h). After activation with MAO, the catalytic system could obtain elastomeric polypropylene. Busico et al. [56] reported complex **26** with very high activity (18.0 × 10^7^ g-PP/mol-Hf·h) for propylene polymerization, which had bulky steric hindrance substituents on the amido and pyridine rings, and the isotactic polypropylene produced by complex **26** had high values of *M*_w_ (above 700 kDa) with narrow MWD. Liu et al. [57] reported bis(pyridine-amido) binuclear titanium complex **27**, which formed a more stable flat structure, providing more space for coordination of the ethylene monomer. The **27**/MAO catalytic system had high catalytic activity (12.4 × 10^4^ g-PE/mol-Ti·h), but because of the binuclear arrangement, the product of HDPE had a wide MWD (10.7). Compared with complex **27**, Zhou et al. [58] reported that zirconium complex **28** with tris(aminopyridinato) ligand had higher activity (14.7 × 10^4^ g-PE/mol-Zr·h). The high Lewis acidity of the metal center combined with the strong basicity of the amide and pyridine ligands as well as the chelating strained *η*^2^-coordination of the amino–pyridine ligand contributed to the increased activity of complex **28**. It was found that the presence of a mono- or multi-aminopyridine ligand complex enables the stabilization of the metal center through the lone nitrogen pair, resulting in diverse complex structures exhibiting high activity for olefin polymerization.

The primary research focus for pyrazolato-based metal complexes is metal complexes with tripyrazolato-based [N,N,N]-tridentate ligands, which have been extensively studied over the years. Cuenca et al. [59] reported that [N,N]-bidentate pyrazolato zirconium complex **29** had low catalytic activity (38.0 × 10^3^ g-PE/mol-Zr·h) for ethylene polymerization and the MWD of the polymer was greater than 57.

Sun et al. [60] reported imidazole-amine hafnium complexes **30a**–**b** and thiazole-amine hafnium complexes **31a**–**31e** for isospecific polymerization of propylene. They found that of all the catalysts screened, complex **30a** exhibited the best combination of activity and stereospecificity. At 130 °C, **30a** still had high activity (78.0 × 10^6^ g-PE/mol-Hf·h). This could be explained by the steric bulk of the aryl substituent at the chiral center at the bridge position; the Hf-N bond length in imidazole-amine hafnium was slightly shorter than thiazole, which led to the higher basicity of the imidazole amine, and the five-membered benzofurazan ring meant that orthometalation may have been more likely to occur, which lessened the unfavorable steric interactions. After the activation of TMA(trimethylaluminum)/[PhNMe_2_H]^+^[B(C_6_F_5_)_4_]^−^, complexes **31a**–**b** had very low activity, and the resulting polypropylene was not highly isotactic. In contrast to **31a** and **31b**, **31c**–**31e** had high activity, yielding polymer with high *M*_w_ and higher crystallinity. Among them, **31d**/TMA/[PhNMe_2_H]^+^[B(C_6_F_5_)_4_]^−^ showed the highest activity (40.9 × 10^7^ g-PE/mol-Hf·h) and *M*_w_ (1320 k). The Hf heterocyclic amido complexes exhibit significant potential and value in olefin polymerization, particularly when modified with diverse heteroaryl groups. These modifications enhance their high temperature resistance, thereby enabling the exploration of copolymerization of higher α-olefins.

**Scheme 3:**
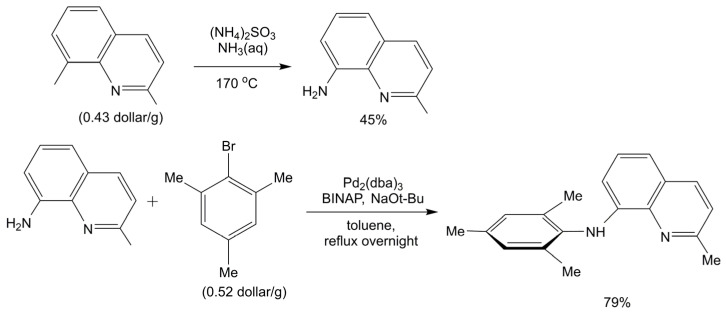
The synthesis of a new route to an aminoquinoline ligand.

Amidoquinoline ligand metal complexes also have excellent thermostability; however, their high synthesis costs limit their application in olefin polymerization. Fontaine et al. [61] reported a novel synthetic pathway to amidoquinoline olefin polymerization catalysts **32a**–**32c**, which involved the utilization of more cost-effective and readily available starting materials. The synthesis of the new route to an aminoquinoline ligand is shown in Scheme 3. After activation with [HNMe(C_18_H_37_)_2_]^+^[B(C_6_F_5_)_4_]^−^, complexes **32a**–**32c** can be applied to ethylene/1-octene copolymerization. At 140 °C, when the molar ratio of 1-octene to ethylene was 2:1, complex **32a** showed high catalytic activity (46.7 × 10^6^ g-copolymer/mol-Hf·h) and incorporation of 1-octene (13.3 mol%) and produced high-molecular-weight copolymer (*M*_w_ = 4.61 × 10^5^ g/mol). However, the polymerization activity and molecular weight of complex **32b** with zirconium were significantly lower than those of **32a**. Additionally, under the same conditions, titanium complex **32c** exhibited complete inactivity. Li et al. [62] reported group 4 metal complexes **33a**–**33d** with an amido-trihydroquinoline ligand that had excellent thermal stability in ethylene polymerization and ethylene/1-octene copolymerization. Complex **33a** with bulk substituents, activated by [Ph_3_C][B(C_6_F_5_)_4_], had high activity (88.8 × 10^6^ g-PE/mol-Hf·h) in ethylene polymerization, and the catalytic systems with different metal centers had their own suitable cocatalysts. Hf complexes produced polymers with higher molecular weights (*M*_w_ above 10^6^ g/mol) in comparison to Zr and Ti complexes by using [Ph_3_C][B(C_6_F_5_)_4_] as a cocatalyst, while Zr complexes produced polymers with high molecular weight with B(C_6_F_5_)_3_ as the cocatalyst (*M*_w_ = 93.1 × 10^4^ g/mol). In addition, complexes **33a** had high 1-octene incorporation (43.5 mol%) in the copolymerization of ethylene and 1-octene, which was higher than complex **32a**. The complexes containing aminoquinoline or its derivative ligands were found to be generally suitable for high-temperature (co)polymerization, primarily due to the enhanced rigidity of the ligand resulting from the presence of aromatic rings. To better illustrate the significant impact of complex structure on catalytic activity and product performance, the (co)polymerization results of some N-heterocyclic ligand complexes with pyridine, pyrazolato, imidazole, quinoline, and trihydroquinoline are shown in Table 6.

## 3. Conclusions and Outlook

Non-metallocene catalysts have been a research focus in the field of olefin polymerization catalysts for the past two decades. Among them, nitrogen-donor-containing bidentate ligands play an important role, especially for [N,N] ligand(s). [N,N] ligand(s) metal complexes, as a series of non-metallocene catalysts with great variety and a wide range of applications, have progressed the development of more new polyolefin materials, such as UHWMPE, olefin copolymers (E/NBE and ethylene/α-olefin) with high insertion rates, and highly isotactic or syndiotactic polypropylene. The catalytic systems mentioned in this paper generally have high catalytic activity for olefin polymerization, but there are also extremes, such as thiazole-amine hafnium complex **31d** with extremely high catalytic activity and pyrazolato zirconium complex **29** with very low catalytic activity. The synthetic raw materials of [N,N] bidentate ligands containing *N*-heterocyclic pyridine amine and quinoline amine are readily accessible at low cost, and the corresponding metal complexes can be easily synthesized. The structure of ligands determines the performance of catalytic systems. In [N,N] ligands, different metal complexes have various properties. The titanium complexes bis(benzamidinate) (**2d**), β-diiminato (**9a**), and amino-containing (**13a**) have higher activity than zirconium complexes that have the same ligand(s), while guanidinato binuclear (**5f**) and pyrrolide-imine zirconium (**21a**) have higher activity than other metal complexes. The thiazole–, imidazole–, and pyridine–amine ligands containing *N*-heterocyclic groups are relatively rare [N,N] bidentate ligands coordinated with hafnium, which exhibit superior properties compared with titanium or zirconium complexes. However, the performance of catalysts is not only affected by metal. In most of the complexes, the steric hindrance and electronic effect of the catalyst system can be changed by modifying the substituents on the ligand skeleton, influencing the activity and thermal stability of the system. However, in non-symmetric guanidinato complexes, the modified ligands have little effect on the catalytic system. The presence of a single metal center is commonly observed in [N,N] non-metallocene catalysts, and binuclear or multinuclear systems are also present, although they may lead to the appearance of multiple active sites, resulting in a wider molecular weight distribution within the polymer. Additionally, the performance of catalysts is also influenced by the choice of solvent and co-catalyst, as well as modifications to the polymerization conditions.

The design of novel catalyst structures and further exploration into catalytic mechanisms will enhance the potential application of [N,N] bidentate non-metallocene catalysts in a wider range of fields. From oligomers to ultrahigh-molecular-weight polymers and atactic to highly isotactic polymers, complexes with different structures can obtain products with different monomer incorporation, molecular weight, and distribution. It is crucial to identify the coordination number between the ligand(s) and metal center and investigate the electronic and steric hindrance on the ligand framework, as well as to screen complex structures with diverse properties. At present, bidentate non-metallocene catalysts exhibit superior activity, thermostability, and adjustability of ligand structure compared with traditional catalysts. Catalysts with exceptional performance have been successfully employed in both industrial and commercial applications. However, when compared with the entirety of non-metallocene catalyst systems, the potential for further enhancement remains. The development of new ligands remains the primary focus as a future direction for advancement, and based on the research into early non-metallocene catalysts, combination with other types of catalysts is also a direction of development.

## Figures and Tables

**Table 1:** Propylene polymerization results of monoamidinate titanium complexes at −60 °C.

Complex	Solvent	[Propene] (mol·L^−1^)	[mm] (%)	[mr] (%)	[rr] (%)	Vicinal Methyl Fraction (%)	Time (h)	Yield (g)	*M*_w_ (×10^4^)	*M*_w_/*M*_n_	Ref.
**1a**	Toluene	5.9	5	42	53	2.5	16	0.40	1.2	1.76	[19]
**1b**	Toluene	5.9	4	41	55	2.3	17	0.63			[19]
**1c**	Toluene	5.9	5	43	52	3.2	17	1.10	1.5	1.71	[19]
**1d**	Toluene	5.9	7	40	53	4.8	16	0.32			[19]
**1e**	Toluene	5.9	39	32	29	1.8	16	0.05			[19]
**1f**	Toluene	5.9	41	39	20	1.7	16	0.05			[19]
**1g**	Toluene	5.9	9	40	51	2.5	16	0.30			[19]

**Table 2:** Polymerization dates of monoamidinate and bis(benzamidinate) complexes ^a^.

Monomer ^b^	Complex	Amount (μmol)	Al:M	Activity ^c^	Temp. (°C)	[mmmm] %	Time (h)	*M*_w_ (×10^4^)	*M*_w_/*M*_n_	Ref.
P	**1a**	20	200	50.0 × 10^1^	−60		16	1.20	1.76	[19]
P	**1c**	20	200	12.9 × 10^2^	−60		17	1.50	1.71	[19]
P	**1c**	50	200	31.3 × 10^3^	20		2			[19]
P	**1a**	50	200	30.0 × 10^3^	20		2			[19]
S	**1a**	50	200	20.0 × 10^2^	50		1			[19]
BD	**1c**	50	200	13.3 × 10^2^	−20		3			[19]
P	**2a**	7.4	250	11.0 × 10^4^	25	86.0		44.00	1.69	[23]
P ^d^	**2a**	7.4	1000	79.0 × 10^4^	25	98.0		8.30	1.42	[23]
P	**2d**	20	1000	28.5 × 10^5^	25	17.0		31.01	2.48	[21]
HD	**2c**	7.9	400	68.0 × 10^2^	25			1.35	2.62	[27]
HD	**2f**	7.2	800	11.0 × 10^4^	50			0.47	1.50	[27]
P	**2g**	15.5	1000	19.0 × 10^2^	25	63.5	3	4.76	6.80	[24]
P	**2k**	15.5	1000	36.0 × 10^2^	25	71.0	3	379.32	2.33	[24]
P	**2l**	15.5	1000	13.7 × 10^3^	25	66.1	3	1.54	2.57	[24]
P	**2m**	15.5	1000	93.0 × 10^2^	25	65.0	3	5.12	5.12	[24]

^a^ The solvent in all of the polymerization was toluene. ^b^ P = propylene, S = styrene, BD = 1,3-butadiene, HD = 1,5-hexadiene. ^c^ g-polymer per mol-M per h. ^d^ solvent was CH_2_Cl_2._

**Table 3:** The polymerization results of β-diiminato complexes ^a^.

Monomer	Complex	Amount (μmol)	Al:M	Pressure (atm)	Temp. (°C)	Time (min)	Activity ^b^	*M*_w_	MWD	H/% ^c^	Ref.
E	**7a**	1	2000	1	30	5	76.8 × 10^5^	50.27 × 10^2^	2.30		[32]
E	**7c**	5	2000	1	30	60	88.0 × 10^2^	13.13 × 10^4^	6.53		[32]
E/H ^d^	**7a**	1	2000	1	30	5	26.1 × 10^6^	15.48 × 10^3^	1.82	32.3	[32]
E/H ^d^	**7c**	5	2000	1	30	60	13.2 × 10^3^	21.13 × 10^4^	13.35	7.4	[32]
E	**8a**	5	1000	10	70	30	10.0 × 10^5^	26.00 × 10^5^	4.10		[34]
E	**8b**	5	1000	10	70	10	11.0 × 10^5^	79.00 × 10^4^	7.20		[34]
E	**9a**	0.1	400	6	40	20	65.0 × 10^5^				[35]
E	**9d**	0.1	400	6	40	20	39.0 × 10^5^				[35]
E/H ^e^	**9a**	0.1	400	6	40	20	51.0 × 10^5^				[35]
E	**10**	5	500	1	50	30	22.4 × 10^4^	12.40 × 10^5^	6.70		[37]

^a^ The solvent for all of the polymerization was toluene. ^b^ g-polymer per mol-M per h. ^c^ Hexene contents in copolymer. ^d^ E = ethylene, H = 1-hexene (5 mL). ^e^ E = ethylene, H = 1-hexene (60 μL).

**Table 4:** The polymerization results of amino-containing complexes ^a^.

Monomer	Complex	Amount (μmol)	Al:M	Pressure (atm)	Temp. (°C)	Time (min)	Activity ^b^	*M*_w_ (g/mol)	MWD	Ref.
E	**13a**	2.7	excess	40	70	45	30.7 × 10^4^			[40]
E	**13b**	3.6	excess	40	70	15	11.2 × 10^4^			[40]
E	**14a**	5	1000	10	70	30	56.6 × 10^3^			[41]
E	**14b**	5	1000	10	70	30	45.0 × 10^3^			[41]
E	**15a**	5	1000	10	70	30	69.2 × 10^4^	25.30 × 10^4^	4.20	[42]
E	**15b**	5	1000	10	70	30	97.6 × 10^4^	41.10 × 10^4^	11.00	[42]
E/H ^c^	**15b**	5	1000	10	70	30	10.3 × 10^5^	33.40 × 10^4^	26.00	[42]
E	**15c**	5	1000	10	70	30	17.3 × 10^5^	33.70 × 10^4^	8.70	[42]
E	**16**	5	1000	10	50	30	48.6 × 10^4^	95.10 × 10^4^	10.08	[43]
E	**17a**	1	2000	4	90	60	18.8 × 10^4^	40.00 × 10^5^	7	[44]
E	**17b**	1	2000	4	90	60	18.5 × 10^4^	90.00 × 10^4^	8	[44]
E/CPE ^d^	**17b**	1	2000	4	50	60	75.0 × 10^5^	50.00 × 10^3^	2.5	[44]
E	**18a**	2	1000	10	30	30	36.0 × 10^3^	20.50 × 10^5^	1.49	[45]
H ^e^	**19a**	47	^f^			30		20.00 × 10^3^	1.30~1.40	[46]
E	**19b**	46	^f^			5	10.2 × 10^4^			[47]

^a^ The polymerization solvent of complex **19b** was C_6_H_5_Cl, and the other polymerization solvents were toluene. ^b^ g-polymer per mol-M per h. ^c^ E = ethylene, H = 1-hexene (6 mL). ^d^ E = ethylene, CPE = cyclopentene (40 vol%) CPE content in the copolymer was 3.5 mol%. ^e^ H = 1-hexene. ^f^ Co-catalyst was [Ph_3_C][B(C_6_F_5_)_4_].

**Table 5:** Polymerization data of complexes **22a**–**22e** ^a^.

M ^b^	Complex	Amount (μmol)	MAO (equiv.)	Pressure (atm)	Temp. (°C)	Time (min)	Activity ^c^	*M*_w_ (kg/mol)	MWD	Ref.
E	**22a**	5	500	10	80	30	40.0 × 10^3^	1760	8,1	[52]
E	**22b**	5	500	10	80	30	52.0 × 10^3^	2800	7.3	[52]
E	**22c**	5	500	10	80	30	56.0 × 10^3^	1980	5.6	[52]
E	**22d**	5	500	10	80	30	96.0 × 10^3^	660	7.7	[52]
E	**22e**	5	500	10	80	30	94.0 × 10^3^	560	7.6	[52]
E/NBE ^d^	**22d**	7.5	500	10	80	10	20.0 × 10^5^	90	6.0	[52]
E/NBE ^e^	**22d**	7.5	500	5	80	10	17.0 × 10^5^	55	4.2	[52]

^a^ The solvent in all of the polymerization was toluene. ^b^ E = ethylene, NBE = norbornene. ^c^ g-polymer/mol-Ti·h. ^d^ Norbornene = 1.8 mol/L, norbornene content = 31.3 mol%. ^e^ Norbornene = 1.8 mol/L, norbornene content = 40.8 mol%.

**Table 6:** The polymerization results of some N-heterocyclic ligand complexes ^a^.

Monomer	Complex	Amount (μmol)	Al:M	Pressure (atm)	Temp. (°C)	Time (min)	Activity ^b^	*M*_w_ (g/mol)	MWD	Ref.
P	**25**		600		25	600	13.1 × 10^3^	11.34 × 10^4^	3.60	[55]
P	**26**	0.5	8 ^c^	6.9	90	3.3	18.0 × 10^7^	^d^	3.20	[56]
E	**27**	5	2000	10	60	30	12.4 × 10^4^	20.30 × 10^4^	10.70	[57]
E	**28**	5	2000	10	50	30	14.7 × 10^4^	29.60 × 10^4^	60.00	[58]
E	**29**	10	1000	4	25	15	38.0 × 10^3^	25.30 × 10^4^	4.20	[59]
P	**30a**				130		78.0 × 10^6^			[60]
P	**30b**		^e^	6.8	110		66.0 × 10^6^	^d^		[60]
P	**31b**		^e^	6.8	110		24.6 × 10^5^	^d^	13.2	[60]
P	**31d**		^e^	6.8	75		40.9 × 10^7^	^d^		[60]
E/O ^f^	**32a**	0.6	10 ^g^	19.6	140	10	46.7 × 10^6^	46.04 × 10^4^	3.38 ^h^	[61]
E/O ^f^	**32b**	2.5	10 ^g^	19.6	140	10	61.0 × 10^5^	28.52 × 10^4^	3.68 ^h^	[61]
E	**33a**	5	^i^	5	100	2	88.8 × 10^5^	14.63 × 10^5^	3.80	[62]
E	**33c**	5	^i^	5	100	2	48.0 × 10^5^	63.00 × 10^4^	2.20	[62]
E	**33d**	5	^i^	5	100	2	30.0 × 10^5^	61.40 × 10^4^	4.50	[62]
E	**33c**	5	^j^	5	100	2	13.8 × 10^6^	93.10 × 10^4^	3.70	[62]
E/O ^f^	**33a**	5	^j^	5	100	2	38.6 × 10^6^	33.80 × 10^4^	2.50 ^h^	[62]

^a^ The polymerization solvent was toluene. ^b^ g-polymer per mol-M per h. ^c^ Co-catalysts were Al(*i*Bu)_2_H and [Me_2_PhNH][B(C_6_F_5_)_4_] (1.1 equiv). ^d^ *M*_w_ = 710 kDa (complex 26), *M*_w_ = 250 k (complex 30b), *M*_w_ = 220 k (complex 31b), *M*_w_ = 1320 k (complex 31d). ^e^ Co-catalysts were TMA and [PhNMe_2_H]^+^[B(C_6_F_5_)_4_]. ^f^ E = ethylene, O = 1-octene (300 g) in complexes 32a and 32b, 1-octene (3 M) in complex 33a. ^g^ Precatalyst: [HNMe(C_18_H_37_)_2_][B(C_6_F_5_)_4_]:MMAO = 1:1.2:10. ^h^ 1-octene incorporation = 13.3 mol% (complex 32a), 1-octene incorporation = 10.4 mol% (complex 32b), 1-octene incorporation = 43.5 mol% (complex 33a). ^i^ Co-catalyst is [Ph_3_C][B(C_6_F_5_)_4_] (1.1 equiv). ^j^ Co-catalyst is [B(C_6_F_5_)_3_] (1.1 equiv).

## Data Availability

Not applicable.
